# Study on risk factors of diabetic peripheral neuropathy and establishment of a prediction model by machine learning

**DOI:** 10.1186/s12911-023-02232-1

**Published:** 2023-08-02

**Authors:** Xiaoyang Lian, Juanzhi Qi, Mengqian Yuan, Xiaojie Li, Ming Wang, Gang Li, Tao Yang, Jingchen Zhong

**Affiliations:** 1 Affiliated Hospital of Nanjing University of Chinese Medicine，Jiangsu Province Hospital of Chinese Medicine, Nanjing, Jiangsu 210029 China; 2 School of Artificial Intelligence and Information Technology, Nanjing University of Chinese Medicine, Nanjing, 210023 Jiangsu China; 3 Jiangsu Health Vocational College, Nanjing, 210036 Jiangsu China; 4 Geriatric Hospital of Nanjing Medical University, Jiangsu Province Official Hospital, Nanjing, Jiangsu 210036 China

**Keywords:** Machine learning, Data analysis, Diabetes, Diabetic peripheral neuropathy

## Abstract

**Background:**

Diabetic peripheral neuropathy (DPN) is a common complication of diabetes. Predicting the risk of developing DPN is important for clinical decision-making and designing clinical trials.

**Methods:**

We retrospectively reviewed the data of 1278 patients with diabetes treated in two central hospitals from 2020 to 2022. The data included medical history, physical examination, and biochemical index test results. After feature selection and data balancing, the cohort was divided into training and internal validation datasets at a 7:3 ratio. Training was made in logistic regression, k-nearest neighbor, decision tree, naive bayes, random forest, and extreme gradient boosting (XGBoost) based on machine learning. The k-fold cross-validation was used for model assessment, and the accuracy, precision, recall, F1-score, and the area under the receiver operating characteristic curve (AUC) were adopted to validate the models’ discrimination and clinical practicality. The SHapley Additive exPlanation (SHAP) was used to interpret the best-performing model.

**Results:**

The XGBoost model outperformed other models, which had an accuracy of 0·746, precision of 0·765, recall of 0·711, F1-score of 0·736, and AUC of 0·813. The SHAP results indicated that age, disease duration, glycated hemoglobin, insulin resistance index, 24-h urine protein quantification, and urine protein concentration were risk factors for DPN, while the ratio between 2-h postprandial C-peptide and fasting C-peptide(C2/C0), total cholesterol, activated partial thromboplastin time, and creatinine were protective factors.

**Conclusions:**

The machine learning approach helped established a DPN risk prediction model with good performance. The model identified the factors most closely related to DPN.

**Supplementary Information:**

The online version contains supplementary material available at 10.1186/s12911-023-02232-1.

## Introduction

The incidence of diabetes has been increasing worldwide in recent years. About 6·7 million people died from diabetes or its complications in 2021 [1]. It is estimated that by 2045, China will have 174 million people with diabetes, ranking first in the world. The burden of diabetes-related complications is expected to increase with the increase in diabetes prevalence. Diabetic peripheral neuropathy (DPN) is the most common microvascular complication of diabetes [2], potentially leading to foot deformity, ulceration, and even amputation. It can also damage the central nervous system and increase the risk of all-cause and cardiovascular mortality [3–6]. Recent studies have found that DPN starts progressing in prediabetes [7]. However, a nerve conduction study could not assess its subtle damage to nerve fibers, resulting in delayed DPN diagnosis, intervention, and treatment administration [8, 9]. Identifying risk factors for DPN is crucial for clinical management.

Studies have identified age, diabetes duration, and glycosylated hemoglobin as risk factors for DPN progression [10, 11], and various other DPN-related factors are still being explored. However, due to differences in evaluation criteria among studies, many contrasting conclusions have been reported [12, 13], resulting in poor clinical applicability of DPN predictors. With the development of science and technology, the establishment of accurate predictive models through machine learning and risk factor acquisition are now applied clinically [14].

Machine learning does not require model structure pre-specification; rather, machine learning searches for the optimal fit within certain constraints. This approach can result in an accurate final prediction model that analyzes the complex interactions between many features [15]. Only a few DPN prediction models have been developed based on machine learning. Considering that DPN prevalence varies among countries [14], the existing DPN prediction models for China are limited by their small sample and single-center nature [16]. Our study aimed to discover the link between laboratory indicators and DPN through machine learning, and help clinicians quickly and accurately predict the risk of developing DPN.

## Methods

### Study population

**Fig. 1:**
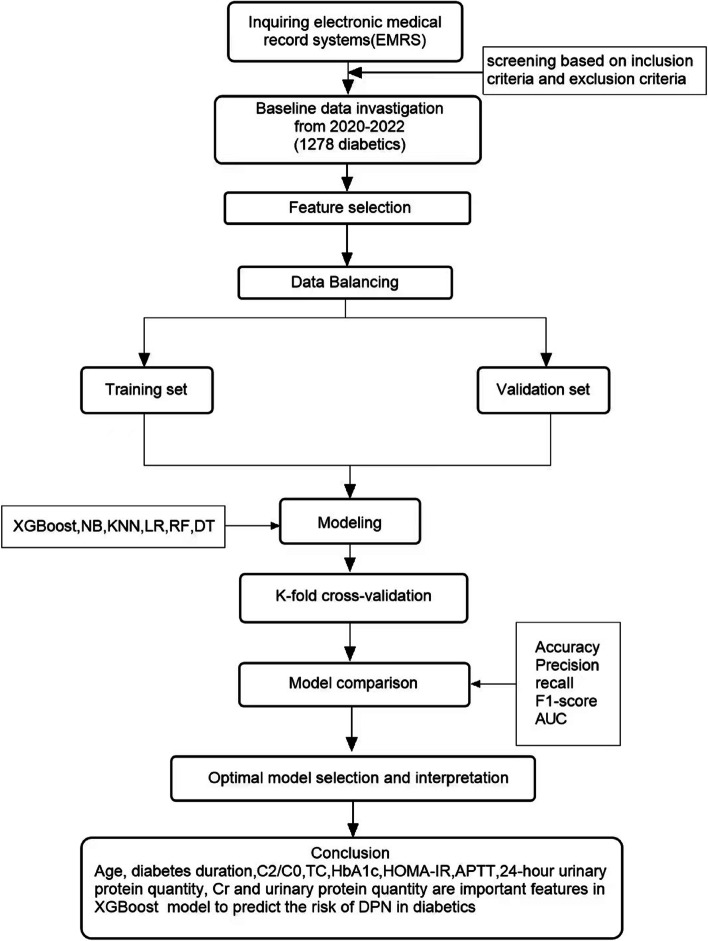
Study design for building a machine learning model to predict diabetic peripheral neuropathy. Abbreviations and definitions: XGBoost, Extreme Gradient Boosting; NB, Naive Bayes; LR, Logistic Regression; KNN, K-Nearest-Neighbor; RF, Random Forest; DT, Decision Tree; K-Fold, K-Fold cross validation; SHAP, SHapley Additive exPlanations

We retrieved the data of 1278 patients with T2DM treated at Jiangsu Provincial Hospital of Traditional Chinese Medicine (*n* = 1093) and Jiangsu Provincial Governmental Hospital (*n* = 185) between February 2020 and July 2022. The data included 192 indicators, including the patient’s basic characteristics, complications, routine blood values, blood biochemistry, immune testing, thyroid function, coagulation function, routine urine values, urine biochemistry, routine stool values, insulin measurement, tumor screening, and sex hormone testing (see Supplementary Table S1).The data were divided into those with and those without DPN according to the nerve conduction test results. See Fig. 1 for the entire research process, including feature selection, data balancing, model construction, model comparison, and optimal model selection and interpretation. This study followed the principles of the Declaration of Helsinki and was approved by the Ethics Committee of Jiangsu Provincial Government Hospitals (2022 Hospital Ethics Review No. 030). All the above data have passed ethical verification.

### Sample size

 In order to achieve better performances of ML models in predicting the risk factors of diabetic peripheral neuropathy in this study, we included all records who fulfilled our inclusion criteria to fully train the models.

### Data extraction

Screening of patient records according to the following inclusion and exclusion criteria.

#### Inclusion criteria

T2DM Patients diagnosed with DPN and had the diagnostic basis of EMG.

#### Exclusion criteria

Patients with neuropathy caused by other factors or patients with intervertebral disc disease, spinal nerve root disease and other neuropathy diseases.

### Data exploration

We invited medical experts to manually select the features with a significant impact on DPN diagnosis, reducing the 192 features in the dataset to 53. The features were selected based on their clinical relevance and previous research and included the patient’s age, weight, glycosylated hemoglobin, blood lipids, and other essential characteristics. Some of these commonly used medical indicators are calculated by other indicators, as shown in the following formulae. Data mining treated the variables as complete (no without values) or incomplete (with missing values). Important incomplete variables were dealt with by deletion, imputation, or no processing. We eliminated ten features whose missing data rate exceeded 30%. After eliminating features, the remaining categorical variables with missing data were completed with the variables’ modes, and the remaining continuous variables were filled with the variables’ mean values. This study included 748 samples with DPN and 530 samples without DPN.

1 $$\mathrm C2/\mathrm C0=\frac{2\;\mathrm{hours}\;\mathrm{postprandial}\;\mathrm C-{\mathrm{peptide}\left(\mathrm{ng}/\mathrm{mL}\right)}}{\mathrm{fasting}\;\mathrm C-\mathrm{peptide}\left(\mathrm{ng}/\mathrm{mL}\right)}$$

2 $$\mathrm{HOMA}-\mathrm{IR}=\frac{\mathrm{fasting}\;\mathrm{plasmaglucose}(\mathrm{mmol}/\mathrm L)\times\mathrm{fasting}\;\mathrm{seruminsulin}(\mathrm{mU}/\mathrm L)}{22\cdot5}$$

3 $$\mathrm{NLR}=\frac{\mathrm{number}\;\mathrm{of}\;\mathrm{neutrophils}(10^9/\mathrm L)}{\mathrm{number}\;\mathrm{of}\;\mathrm{lymphocytes}(10^9/\mathrm L)}$$

### Feature selection and data balancing

Feature selection, critical in feature engineering, can filter out highly correlated features to improve model performance and reduce training time. Feature selection can be divided into the filtering, wrapping, and embedding methods. The embedding feature selection method employed in this study uses a machine learning model to automatically select features and integrate them into the training process.

We balanced the data as most machine learning algorithms do not work well with unbalanced datasets. For unbalanced data, over-sampling, under-sampling or both can be used to achieve positive and negative sample balance. The SMOTETomek method [17, 18] was used for data balancing. It is a composite method that performs an over-sampling operation on a large proportion of samples and an under-sampling operation on a small proportion of samples.

### Research technology

Statistical analysis was performed using IBM SPSS Statistics for Windows, Version 27.0 (IBM Corp., Armonk, NY, USA). We used the Kolmogorov-Smirnov test to assess the continuous variables and the Chi-Squared test to assess the categorical variables (see Supplementary Table S2 and S3). As the continuous variables were not normally distributed, they were compared by the Mann-Whitney *U* test. Feature selection and data preprocessing, balancing, modeling, and evaluation were performed using Python Software Foundation. Python Language Reference, version 3.9. Available at http://www.python.org.

We divided the data into a 7:3 ratio of training set and test set, using the training set to train the prediction model and the test set for the model evaluation. Six machine learning algorithms were used to build the prediction models, including logistic regression, k-nearest neighbor, decision tree, naive Bayes, random forest, and extreme gradient boosting (XGBoost). We used the open-source package sklearn 0.24.2 for model realization and evaluation [19]. Model performances were assessed by the indicators’ accuracy, precision, recall, F1-score, confusion matrix, and the area under the receiver operating characteristic curve (AUC) under 10-fold cross-validation.

### Model interpretation

The interpretation of the model is a very important step that helps one to understand the process of model classification. The SHapley Additive exPlanations(SHAP) originated from cooperative game theory and have a solid theoretical foundation [20]. The method is a model-independent solution to model interpretability. We synthetically selected the best-performing model, and used SHAP to calculate the marginal contribution of features to explain the model output, identify significant features in the various classifications, and indicate whether they were positively or negatively correlated.

## Results

### Participants

We used the collected data of 1278 diabetic patients for modeling of machine learning, all patients have completed EMG results. Nerve conduction abnormalities involving one or more nerves was defined as nerve injury, grouped according to the patient’s nerve conduction outcome. 748 (58.53%) of which did and 530 (41.47%) did not develop DPN. The description of the general situation of the study subjects is presented in Supplementary Table S3.

### Feature selection

The 43 variables included in this study comprised 34 continuous variables and nine categorical variables. We used the embedded method and random forest as primary learners to further filter the features and select 16. Supplementary Table S4 presents the weights of these 16 features.

As shown in Table 1, age, alanine aminotransferase, albumin, total bilirubin, urea, total cholesterol, glycosylated hemoglobin, activated partial thromboplastin time (aPTT), 24-h urine protein quantification, urine protein concentration, diabetes duration, neutrophil-to-lymphocyte ratio, and the homeostatic model assessment of insulin resistance (HOMA-IR) index were statistically significant (*P<*0·05).

**Table 1:** Single factor analysis of DPN selection variables

Characteristic	NDPN	DPN	*P*value^a^
Age(years)	56·0(46·0–65·0)	64·50(55·0–72·0)	**< 0·001**
Alanine aminotransferase (U/L)	20·0(14·0–22·0)	17·0(13·0–24·0)	**< 0·001**
Albumin(g/L)	41·9(39·6–43·8)	40·8(38·4-–43·3)	**< 0·001**
Total bilirubin(µmol/L)	10·4(7·9–13·8)	9·9(7·3–13·0)	**0·005**
Urea(mmol/L)	5·3(4·4–6·4)	5·8(4·7–7·1)	**< 0·001**
Creatinine(µmol/L)	66·6(56·0–77·8)	66·1(56·0–80·1)	0·319
Uric acid(µmol/L)	317·0(269·0–393·0)	318·0(262·0–377·0)	0·231
Total Cholesterol(mmol/L)	4·9(3·9–5·3)	4·4(3·6–5·3)	**0·012**
Glycated hemoglobin(mg/dl)	8·0(6·8–9·6)	8·8(7·3–10·5)	**< 0·001**
Activated partial thromboplastin time(s)	36·0(33·4–38·9)	35·0(32·8–37·6)	**< 0·001**
Urine protein quantity(mg/L)	39·0(24·0–75·0)	61·0(32·0–146·0)	**< 0·001**
24 h urine protein quantity(mg/24 h)	80·5(44·5–128·5)	109·0(60·0–263·0)	**< 0·001**
Diabetes duration(years)	5·0(2·0–11·0)	10·0(4·0–18·0)	**< 0·001**
C2/C0	3·3(2·4–4·6)	2·9(1·9–4·1)	**< 0·001**
NLR	1·8(1·3–2·6)	2·1(1·6–3·0)	**< 0·001**
HOMA-IR	2·7(1·6–4·4)	3·1(1·7–5·4)	**0·011**

^a^Statistics: Mann-Whitney U Test for continuous variable comparisons; *P* < 0.05 are in bold

### Data balancing

The data distribution before and after balancing is shown in Table 2.

**Table 2:** Comparison of positive and negative samples before and after data balance

	DPN	NDPN
Before sampling	748	530
After sampling	700	700

*DPN* with diabetic eripheral neuropathy, *NDPN* without diabetic eripheral neuropathy

### Modeling and evaluation

The evaluation results of the six machine learning algorithms are shown in Table 3. The results showed that XGBoost had the best accuracy (0·753 ± 0·032), recall (0·721 ± 0·050), and F1-value (0·744 ± 0·036). K-nearest neighbor showed the highest precision (0·858 ± 0·070) but performed poorly in the other indicators.

**Table 3:** Comparison of classification results of different models (mean ± std)

Algorithm	Accuracy	Precision	Recall	F1-score
LR	0·679 ± 0·052	0·687 ± 0·056	0·659 ± 0·062	0·672 ± 0·056
KNN	0·674 ± 0·039	**0**·**858 ± 0**·**070**	0·419 ± 0·073	0·559 ± 0·070
DT	0·682 ± 0.032	0·695 ± 0.032	0·648 ± 0·067	0·669 ± 0·042
NB	0·590 ± 0·029	0·784 ± 0·087	0·253 ± 0·061	0·378 ± 0·071
RF	0·736 ± 0·021	0·769 ± 0·026	0·677 ± 0·040	0·719 ± 0·027
XGBoost	**0·746 ± 0·041**	0·765 ± 0·040	**0·711 ± 0·066**	**0·736 ± 0·050**

The best results are in bold

*XGBoost* Extreme Gradient Boosting, *NB *Naive Bayes, *LR *Logistic Regression, *KNN *K-Nearest-Neighbor, *RF *Random Forest, *DT *Decision Tree

**Fig. 2:**
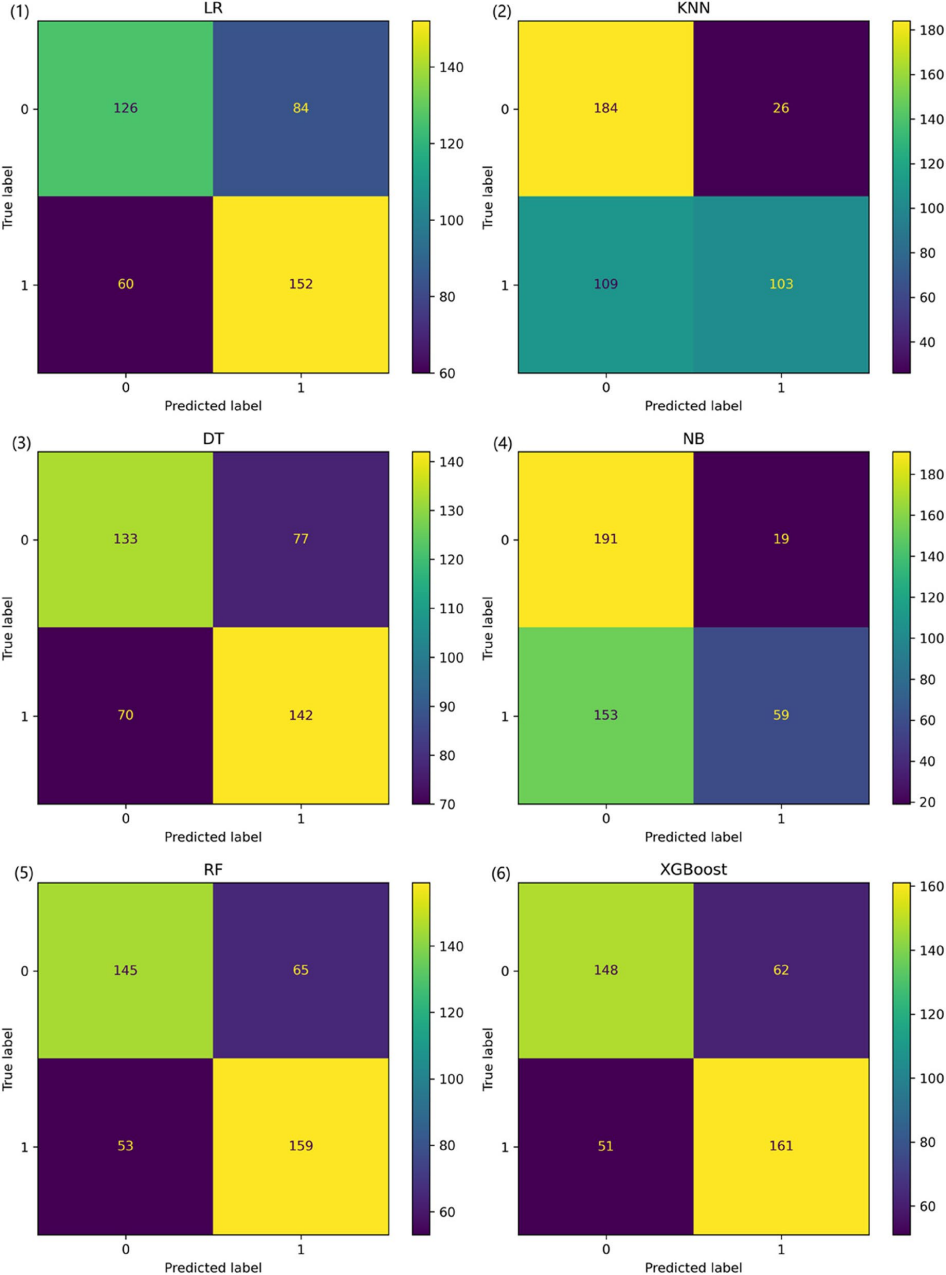
Model classification confusion matrix. XGBoost, Extreme Gradient Boosting; NB, Naive Bayes; LR, Logistic Regression; KNN, K-Nearest-Neighbor; RF, Random Forest; DT, Decision Tree

The confusion matrix serves as a formalized method for evaluating machine learning models, reflecting the results presented in Table 3. As can be easily deduced from Fig. 2, the overall performance of the Random Forest and XGBoost models is significantly superior to that of the other models, with the XGBoost model having a slight edge over the Random Forest model. Specifically, the XGBoost model slightly outperforms the Random Forest model in terms of accuracy, precision, and recall metrics.

**Fig. 3:**
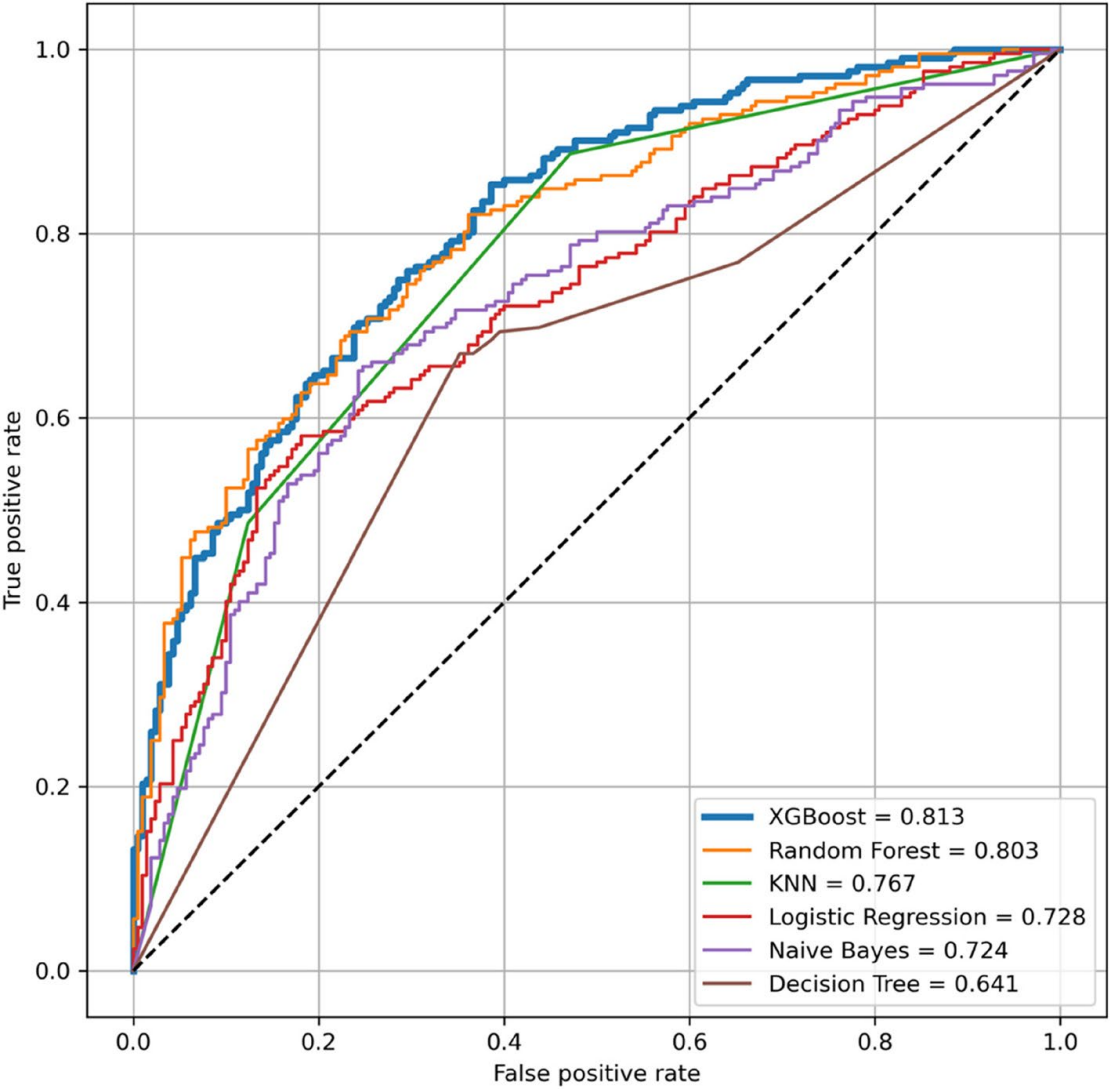
ROC curves for different classification models. XGBoost, Extreme Gradient Boosting; NB, Naive Bayes; LR, Logistic Regression; KNN, K-Nearest-Neighbor; RF, Random Forest; DT, Decision Tree; ROC curve,receiver operating characteristic curve

The performance of the six machine learning algorithms in predicting DPN is shown in Fig. 3. The AUC of XGBoost (0·818) was the largest, followed by random forest (0·804). The decision tree model had the smallest AUC (0·636).

Descriptive statistics of AUC values for different models were shown in Table 4. Due to the small sample size and non-normal distribution of AUC, we conducted a Wilcoxon signed-rank test on the XGBoost model’s and other models’ AUC values. The AUC values of XGBoost were statistically significantly different from those of LR, KNN, NB, and DT, but not significantly different from RF, as shown in Table 5. Cross-validation results were often used for model selection, and based on the average values of various metrics in Table 3, we have chosen XGBoost.

**Table 4:** Descriptive Statistics of AUC for Different Models

	Min	Q1	Median(Q2)	Q3	Max	*P* value
XGBoost	0**·**679	0**·**709	0**·**764	0**·**775	0**·**801	0**·**074
RF	0**·**686	0**·**732	0**·**736	0**·**748	0**·**765	0**·**203
LR	0**·**586	0**·**675	0**·**683	0**·**720	0**·**737	**0·040**
KNN	0**·**607	0**·**650	0**·**671	0**·**698	0**·**739	0**·**853
NB	0**·**543	0**·**568	0**·**589	0.614	0**·**634	0**·**547
DT	0**·**636	0**·**663	0**·**679	0**·**690	0**·**759	0**·**299

*P* < 0.05 are in bold

Performing a Shapiro-Wilk test for normality on the AUC values from 10-fold cross-validation

*XGBoost *Extreme Gradient Boosting, *NB *Naive Bayes, *LR *Logistic Regression, *KNN *K-Nearest-Neighbor, *RF *Random Forest, *DT *Decision Tree

**Table 5:** Significance Testing between XGBoost and Other Models

	RF	LR	KNN	NB	DT
*P* value	0**·**375	**0·002**	**0·002**	**0·002**	**0·006**

*P* < 0.05 are in bold

*XGBoost *Extreme Gradient Boosting, *NB *Naive Bayes, *LR *Logistic Regression, *KNN *K-Nearest-Neighbor, *RF *Random Forest, *DT *Decision Tree

**Fig. 4:**
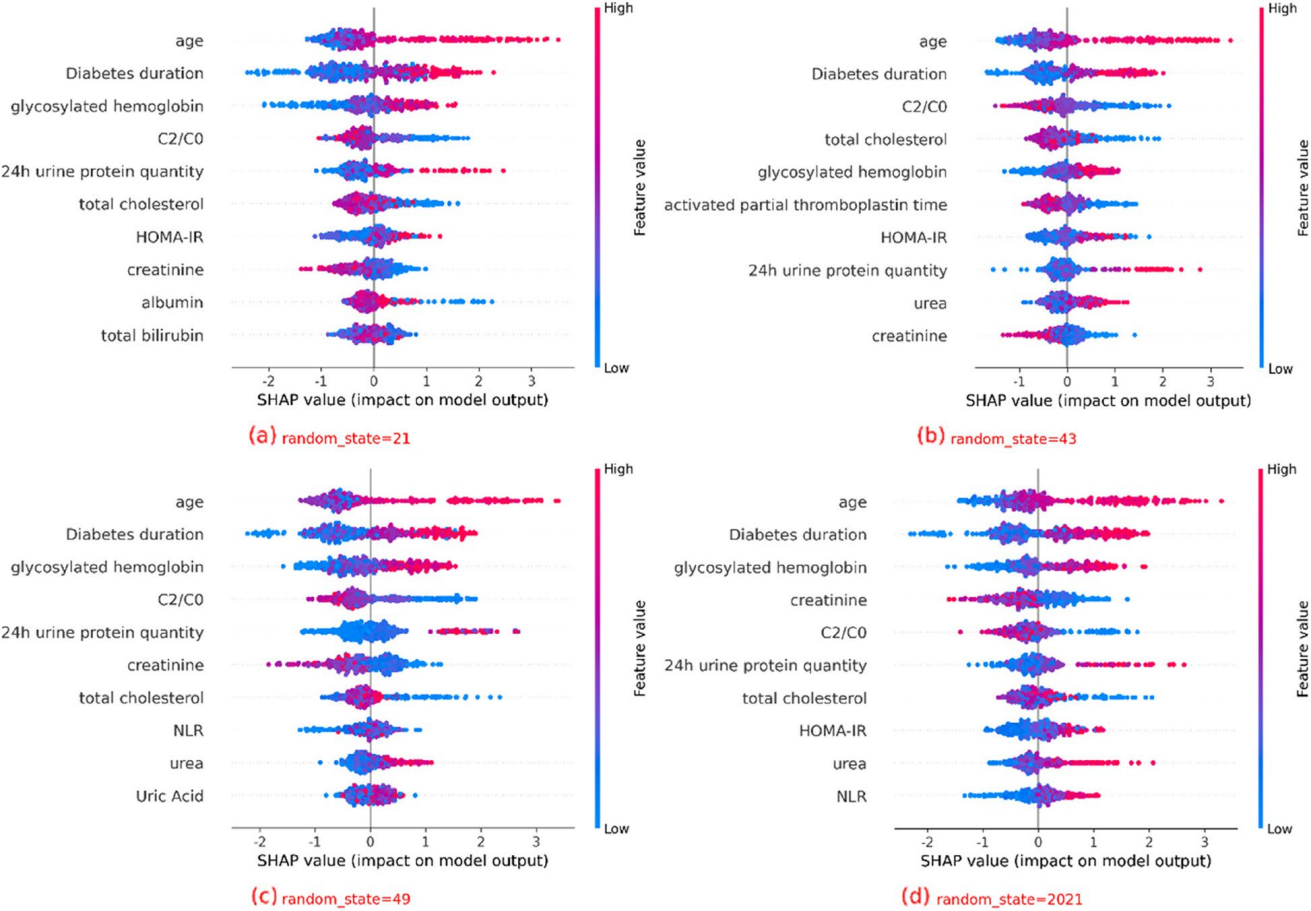
Comparison of XGBoost Model Interpretations using SHAP across Different Dataset Splits

A comprehensive performance analysis of the six models indicated that XGBoost was the optimal model for predicting DPN. We used SHAP to elucidate the relationship between the features and the output of the XGBoost model. From Fig. 4, we can get the top 10 indicators that have the greatest impact on classification, all of which *P* < 0̵·05. Figure 4 shows that age, disease duration, C2/C0, and total cholesterol were essential features for DPN prediction by XGBoost and significantly impacted the classification results. Age, disease duration, glycated hemoglobin, insulin resistance (IR) index, 24-h urine protein quantification, and urine protein concentration were risk factors, while C2/C0, total cholesterol, aPTT, and creatinine were protective factors.

## Discussion

DPN is the most common complication of diabetes in China, in addition to cardiovascular and cerebrovascular diseases. DPN is often neglected in its early stages because nerve conduction studies cannot detect small fiber lesions. The nerve damage caused by DPN is irreversible by the time the disease is fully established. Therefore, it is very important to identify the clinical indicators, predictors, and risk factors of DPN. This study built a DPN prediction model based on XGBoost through machine learning. The high recall rate of the model shows that it has good reliability.

The developed model has several advantages. First, data from two medical centers in Nanjing, China, were used for analysis. DPN diagnosis in all patients followed the nerve conduction study classification, and their pathological data were tested by standardized laboratory techniques, to ensuring the accuracy of the variables in the prediction model. Second, unbalanced datasets make models highly dependent on some specific indicators, leading to over- or underfitting. We used the SMOTETomek method to solve this problem. Third, we constructed multiple classical machine learning DPN prediction models. We determined that the XGBoost model was the best after comparing the models by 10-fold cross-validations. Fourth, the SHAP method was used to explain the relationship between the input features and the output variables of the XGBoost model, ranking the features according to their contribution to the model output. We found several important indicators highly correlated with the progression to DPN. These are highly relevant since the key indicators of disease diagnosis were objectively extracted from real clinical data.

Previous studies have attempted to build predictive models of DPN. Wu et al. [16] established four predictive nomographs and selected the optimal model, but the Toronto Clinical Neuropathy Scoring System score suggested the diagnosis groups included false positives. Kazemi et al. [21] built a DPN prediction model based on multicategory support vector machine; however, by directly selecting the research model, they could not compare it to others, possibly reducing the model’s accuracy. Baskozos et al. [22] used the DOLORisk project dataset, applied machine learning to classify painful and painless DPN, built models, and identified predictive factors. Although their study used one of the largest and most comprehensively phenotyped cohort of people with DPN, its disadvantage was in its uncertain data quality. Metsker et al. [23] used EMRS data to build various machine-learning models for DPN. They found the most effective method for each research task to ensure the high accuracy of the research results. Therefore, it would be useful to build a better DPN prediction model using well-structured, large, and balanced datasets. The previously determined risk factors of DPN include age, diabetes duration, glycosylated hemoglobin, 24-h urine protein quantification, and urine protein concentration [24], consistent with our results. Our research has additional new findings.

The C2/C0 index represents a protective factor of DPN. C2/C0 reflecting the secretory function of pancreatic β cells. The higher the C2/C0 value represents the higher increase in C-peptide after meals. C-peptide is a polypeptide released by pancreatic β cells into the blood at a concentration equal to that of insulin. Laboratory studies have shown that C-peptide can increase endothelial nitric oxide synthase to improve endothelial function, blood flow, and neural function [25]. It can also improve neural function and structural abnormalities by increasing the activity of nerve Na+/K + ATPase and reducing Na + retention [26]. Furthermore, it can improve the gene expression of nerve growth factor, insulin-like growth factor-1, and neurotrophin-3 receptors correcting the mRNA and protein expression of neurofilament and tubulin and normalizing the abnormal phosphorylation of neurofilament [27, 28]. Clinical studies of T1DM showed that C-peptide has a good neuroprotective effect [29]. As T2DM is related to IR, changes in insulin and C-peptide concentrations will occur with the development of the disease. Therefore, there is no clear clinical evidence of the role of C-peptide in T2DM. Most patients in our cohort had T2DM. C2 and C0 were correlated with DPN when we started analyzing the data, but they were not significant enough. Their importance became apparent once they were combined into an index. Our finding confirms the neuroprotective effect C-peptide has in patients with diabetes and further explains the relationship between the secretion function and health of pancreatic β cells and DPN.

Total cholesterol protects against DPN in patients with diabetes. Total cholesterol is the sum of HDL-C, LDL-C, and the small amount of free cholesterol. Cholesterol is closely related to nerves and is an indispensable resource for myelin sheath development [30]. The myelin sheath can ensure the rapid transmission of nerve impulses and maintain normal nerve function in the peripheral nervous system (PNS) [31]. The myelin sheath in the PNS is mainly formed by a repeated wrapping of the Schwann cell membrane around the axon. Most of the cholesterol required to form the myelin sheath is synthesized in the endoplasmic reticulum of the neuronal cell body. However, when a very long axon is damaged away from the cell body, the Schwann cells need to take cholesterol from the circulation to form the myelin sheath [32, 33]. It was suggested that lower serum cholesterol levels might hamper peripheral nerve regeneration [34]. It was proposed that nerve swelling due to changes in Schwann cell lipid components for lack of cholesterol affects axon regeneration after nerve injury [33, 35]. Furthermore, the effect of daily medication on the lipid profile in patients with diabetes should not be ignored. Most patients with diabetes are treated with insulin, which can increase the high-density lipoprotein cholesterol (HDL-C) level, but not the low-density lipoprotein cholesterol (LDL-C) level [36]. Metformin, a commonly used clinical hypoglycemic drug, can reduce LDL-C [37]. It was shown that LDL-C might cause nerve damage [32, 38, 39] while HDL-C protects against it [40]. HDL-C or LDL-C alone did not show a strong correlation with DPN in this study due to the diverse medications used by the patients. However, it can be assumed that changes in the lipid profile after diabetes medication use might affect the nerves. In addition to hypoglycemic drugs, the intake of statins and the related reduction in serum cholesterol level are also associated with accelerated deterioration of neurological symptoms, microvascular injury, and peripheral nerve fiber injury [34, 41, 42]. But in some studies, hyperlipidemia is a risk factor for DPN in patients with T2DM [43]. In clinical practice, most patients need medications to control blood lipids status. Consequently, a lingering question is whether we can predict DPN development based on the proportion of cholesterol in blood lipids, considering the use of drugs to control blood lipid within a certain range.

HOMA-IR is a predictor of DPN in patients with diabetes. It is an index calculated using fasting blood glucose and insulin [44]. HOMA-IR is a good indicator of the degree of IR in the body and has been used in large-scale clinical and epidemiological studies. IR is the main internal environment state in patients with T2DM. Neuronal IR can lead to low insulin signaling and induce DPN progression. IR reduces the Akt signaling transduction by destroying insulin signaling in Schwann cells of the PNS [45]. Alterations in the Akt signaling pathway affect the neuronal mitochondrial function and lead to the subsequent increase in oxidative stress [46]. Glucose can mediate oxidative stress and promote the progression of DPN by inducing mitochondrial biogenesis and fission [47]. Therefore, the disruption to insulin signaling induced by IR makes the PNS neurons more susceptible to metabolic damage. Moreover, Akt regulates the myelination of the PNS nerve fibers by activating Rac1 to enhance membrane encapsulation and synthesizing myelin protein through mammalian target of rapamycin complex 1 [48, 49]. The reduction in Akt signaling transduction caused by IR impairs myelination and enhances DPN progression. IR was associated with DPN in laboratory studies [50], but related clinical data analysis is rare. This study was the first to use the HOMA-IR index as an indicator and explored the correlation between IR and DPN in clinical data.

Our model indicated that aPTT and creatinine were protective factors of DPN. aPTT represents the coagulation ability in the body; the lower the value, the more likely there is a hypercoagulable state. Therefore, it is possible that the nerves have a better blood supply when the body is not in a hypercoagulable state, and the better blood supply delays the progress to DPN. However, considering the obstacles to coagulation in patients with diabetes and the few related studies, this conclusion remains to be explored. Creatinine was mostly associated with diabetic nephropathy in studies on diabetic complications. Its association with DPN should be further explored as there are too few studies on the topic.

The results obtained by the model helped better understand the importance of each feature to the model’s prediction. Among the indicators detected by the model, the ten most closely related to DPN were age, diabetes duration, C2/C0, total cholesterol, glycosylated hemoglobin, HOMA-IR index, aPTT, 24-h urine protein quantification, creatinine, and urine protein concentration. The high correlation between age, diabetes duration, and DPN further highlighted the importance of early intervention to prevent complications in patients with diabetes.

Our study had several limitations that must be considered. First, all participants had diabetes, so we only explored diabetes-related indicators. The prediction results could have been different if we had included a control group of healthy individuals. Second, we lack the collection and analysis of patients’ subjective description. These will need to be addressed in future research. Third, all the indicators in this study were continuous variables, and the data analysis may be segmental. For example, the protective effects of aPTT and creatinine on DPN should be restricted to a certain threshold range. Future studies should focus on the impact of indicators within different thresholds. Fourth, the prediction model data came mostly from Nanjing, Jiangsu Province. The applicability of our results to other regions remains to be verified.

In conclusion, we established a DPN risk prediction model, which showed good performance. Through the model, we identified the factors most closely related to DPN. Our team will address the existing problems and strive to establish a better DPN prediction model through future research to help doctors quickly and accurately judge the corresponding prognosis for improved, reliable, and convenient personalized treatment and management of patients with diabetes.

## Supplementary Information

**Additional file 1: Supplementary Table S1.** All the features in the raw data.

**Additional file 2: Supplementary Table S2.** Kolmogorov-Smirnov test for continuous variables.

**Additional file 3: Supplementary Table S3. **Patient demographics and univariate analysis

**Additional file 4: Supplementary Table S4.** Feature weights in embedded feature selection.

**Additional file 5: Supplementary Table S5.** Hyperparameters combinations in optuna of the 6 models.

## Data Availability

The data used to support the findings of this study are available from the corresponding author upon request.

## Abbreviations

DPN: Diabetic peripheral neuropathy
C2/C0: The ratio between 2-h postprandial C-peptide and fasting C-peptide
NDPN: Non-diabetic peripheral neuropathy
EMRS: Electronic medical record system
LR: Logistic regression
KNN: K-nearest neighbor
DT: Decision tree
NB: Naive bayes
RF: Random forest
XGBoost: Extreme gradient boosting
SHAP: Shapley additive explanation
TN: True negative value
FP: False positive value
FN: False negative value
TP: True positive value
NF: Neurofilament
T1DM: Type 1 diabetes mellitus
T2DM: Type 2 diabetes mellitus
HDL-C: High-density lipoprotein cholesterol
LDL-C: Low-density lipoprotein cholesterol
HOMA-IR: The homeostatic model assessment of insulin resistance
PNS: Peripheral nervous system
IR: Insulin resistance
aPTT: Activated partial thromboplastin time
