# Supplementary material for: Study on risk factors of diabetic peripheral neuropathy and establishment of a prediction model by machine learning
Source: BMC Med Inform Decis Mak. 2023 Aug 2;23:146. doi: 10.1186/s12911-023-02232-1 (PMC10394817; doi:10.1186/s12911-023-02232-1)
Supplement: Supplementary file 1 — Additional file 1: Supplementary Table S1. All the features in the raw data. [file 12911_2023_2232_MOESM1_ESM.docx]

Supplementary Table S1**.** All the features in the raw data.

| **Category** | **Indicators** |
| --- | --- |
| Basic information | Stream number, Name, Case number, Gender, Age, Date of admission, Height, Diabetes duration |
| Type of disease | Diabetic nephropathy(DN), Diabetic retinopathy(DR), Hyperlipidemia(HLD), Hypertension(HTN), Atherosclerosis |
| Blood routine | Ultrasensitive C-reactive protein(hs-CRP), White blood cell count(WBC), Erythrocytes, Hemoglobin(Hb), Platelets, Neutrophil percentage, Lymphocytes percentage, Monocytes percentage, Eosinophil percentage, Basophil percentage, Neutrophil absolute value, Lymphocyte absolute value, Monocyte absolute value, Eosinophil absolute value, Basophil absolute value, Erythrocyte Specific Volume(ESV), Mean red blood cell volume(MCH), Mean hemoglobin content(MCHC), Mean hemoglobin concentration, erythrocyte distribution width coefficient of variation (RDW-CV), erythrocyte distribution width standard deviation (RDW-SD), Mean platelet volume(MPV) |
| lood Biochemistry | Aspartate aminotransferase(AST), alanine aminotransferase(ALT), Total protein(TP), Albumin(ALB), Globulin(GLOB), Albumin/globulin ration(A/G), Prealbumin(PA), Alkaline phosphatase(ALP), Gamma-glutamyl transferase(GGT), Total bilirubin(TBIL), Direct bilirubin(DBIL), Indirect bilirubin(IBIL), Total bile acids(TBA), a-L-rhabdosome(α-L-R), Cholinesterase(CHE), Adenosine deaminase assay(ADA), Serum transferrin(TF), Glutathione reductase(GR), Glutamate dehydrogenase(GDH), Creatine kinase(CK), Lactate dehydrogenase(LDH), Creatine kinase isoenzyme(CK-MB), α-hydroxybutyric acid(α-HBA), Urea, Creatinine, Glucose(GLU), Potassium(K), Sodium(Na), Chlorine(Cl), Calcium(Ca), Serum bicarbonate(HCO3-), Uric acid, Phosphorus(P), Magnesium(Mg), Amylase(AMY), Lipase(LIP), Serum acid phosphatase(ACP), Tartaric acid inhibited acid phosphatase(TRAP), Nitric oxide(NO), Retinol Binding Protein(RBP), Cystatin C(Cys-C), Total cholesterol(TC), Triglycerides(TG), High-density lipoprotein cholesterol(HCL-C), Apolipoprotein A1(ApoA1), Apolipoprotein B(ApoB), Apolipoprotein E(ApoE), Low-density lipoprotein cholesterol(LDL-C), Lipoprotein a(Lp(a)), Glycated hemoglobin(HbA1c), Glycated serum protein(GSP) |
| Immunological tests | Immunoglobulin G(IgG), Immunoglobulin A(IgA), Immunoglobulin M(IgM), Complement C3, Complement C4, Anti-O antibodies, Rheumatoid Factor(RF), C-reactive protein(CRP) |
| Thyroid function | Thyroid stimulating hormones(TSH), Triiodothyronine(T3), Thyroxine (T4) TT4, Free triiodothyronine(FT3), Free thyroxine (FT4), Antithyroglobulin Antibodies , Anti-thyroid peroxidase(anti-TG), Antibody assay Thyroglobulin(anti-TPO), Anti-thyroglobulin antibodies(anti-TG), Parathyroid hormone(PTH) |
| Coagulation | Prothrombin time (PT), International Normalized Rate (INR), Prothrombin time activity, Activated partial thromboplastin time (APTT), Activated partial thromboplastin ratio, Fibrinogen, Prothrombin time(PT), D-dimer, Fibrin(pro)degradation products, Plasma antithrombin |
| Urine Routine | Urinary Retinol Binding Protein(uRBP), Trace protein/creatinine, Urinary Complement C3(uC3), Urinary alpha 2-macroglobulin(uA2M), Urinary alpha1-microglobulin(uA1M), Microalbumin, Urinary creatinine(uCr), Color, Transparency, Occult blood, Hemameba, Bilirubin, Nitrite, Vitamin C, Urobilinogen, Ketone bodies, Urine protein, Glucose, pH, Specific Gravity, Erythrocyte count, Leukocyte count, Leukocyte mass, Squamous Epithelial Cells(SEC), Non-squamous epithelial cells(NSEC), Bacteria, Clear tubular, Granular tubular, Erythrocyte tubular, Leukocyte tubular, Waxy tube, Cellular tubular, Mucus threads, Unclassified crystals, Saccharomyces cerevisiae |
| Urine biochemistry | Urine protein quantity, 24-hour urine protein quantity, 24-hour urine volume |
| Fecal routine | Color, Characteristic, Erythrocytes, Pus cells, Fecal parasite, Fat globule, Charcot-Leyden crystals, Occult blood, Mycobacteria |
| Insulin determination | Fasting C-peptide, Fasting insulin, C-peptide 30 minutes postprandial, 30 minutes post-prandial insulin, C-peptide 60 minutes postprandial, 60 minutes postprandial insulin, C-peptide 120 minutes postprandial, 120 minutes postprandial insulin, C2/C0 |
| Tumor screening | Alpha-fetoprotein(AFP), Carcinoembryonic antigen(CEA), Ferritin, Glycoantigen 50(CA 50), Glycoantigen 242(CA242), Glycoantigen 199(CA 19-9), Glycoantigen 724(CA 724), Neurospecific enolase(NSE), Prostate-specific antigen(PSA), Cytokeratin 19 fragment(CYFRA 21-1), Free prostate-specific antigen(free PSA) |
| Sex Hormone Test | Testosterone, Estradiol, Luteinizing hormone(LH), Follicle-maturation stimulating hormone(FSH), Progesterone, Serum lactogen(SL) |
| Corresponding indicator arithmetic ratio | Neutrophil-to-lymphocyte ratio(NLR), Platelet-to-lymphocyte ratio(PLR), Homeostatic model assessment of insulin resistance(HOMA-IR) |
