# Supplementary material for: Study on risk factors of diabetic peripheral neuropathy and establishment of a prediction model by machine learning
Source: BMC Med Inform Decis Mak. 2023 Aug 2;23:146. doi: 10.1186/s12911-023-02232-1 (PMC10394817; doi:10.1186/s12911-023-02232-1)
Supplement: Supplementary file 2 — Additional file 2: Supplementary Table S2. Kolmogorov-Smirnov test for continuous variables. [file 12911_2023_2232_MOESM2_ESM.docx]

**Supplementary Table S2.** Kolmogorov-Smirnov test for continuous variables.

| Characteristic | NDPN^a^ | DPN |
| --- | --- | --- |
| Age | <0·001 | <0·001 |
| Height | <0·001 | <0·001 |
| Weight | <0·001 | <0·001 |
| White blood cell count ^b^ | <0·001 | <0·001 |
| Hemoglobin | **0·200** | <0·001 |
| Monocyte absolute value | <0·001 | <0·001 |
| Eosinophil absolute value | <0·001 | <0·001 |
| basophil absolute value | <0·001 | <0·001 |
| Mean red blood cell volume | <0·001 | <0·001 |
| Aspartate aminotransferase | <0·001 | <0·001 |
| Alanine aminotransferase | <0·001 | <0·001 |
| Albumin | <0·001 | <0·001 |
| Total bilirubin | <0·001 | <0·001 |
| UREA | <0·001 | <0·001 |
| Creatinine | <0·001 | <0·001 |
| Serum bicarbonate | 0·032 | **0·200** |
| Uric acid | <0·001 | <0·001 |
| Lipase | <0·001 | <0·001 |
| Total cholesterol | <0·001 | <0·001 |
| Triglycerides | <0·001 | <0·001 |
| High-density lipoprotein cholesterol | <0·001 | <0·001 |
| Low-density lipoprotein cholesterol | 0·012 | <0·001 |
| Glycated hemoglobin | <0·001 | <0·001 |
| Thyroid stimulating hormones | <0·001 | <0·001 |
| Prothrombin time | <0·001 | <0·001 |
| Activated partial thromboplastin time | <0·001 | <0·001 |
| Urine protein quantity | <0·001 | <0·001 |
| 24h urine protein quantity | <0·001 | <0·001 |
| Diabetes duration | <0·001 | <0·001 |
| C2/C0 | <0·001 | <0·001 |
| NLR | <0·001 | <0·001 |
| PLR | <0·001 | <0·001 |
| HOMA-IR | <0·001 | <0·001 |

^a^NDPN: No diabetic peripheral neuropathy; ^b^White blood cell count: White blood cell count in blood routine; P>0.05 are in bold.
