# Supplementary material for: Study on risk factors of diabetic peripheral neuropathy and establishment of a prediction model by machine learning
Source: BMC Med Inform Decis Mak. 2023 Aug 2;23:146. doi: 10.1186/s12911-023-02232-1 (PMC10394817; doi:10.1186/s12911-023-02232-1)
Supplement: Supplementary file 3 — Additional file 3: Supplementary Table S3. Patient demographics and univariate analysis [file 12911_2023_2232_MOESM3_ESM.docx]

**Supplementary Table S3·** Patient demographics and univariate analysis·

| Characteristic | NDPN^a^(N=530) | DPN(N=748) | P value^b^ |
| --- | --- | --- | --- |
| **Basic information** | | | |
| Gender (%) | | | 0·717 |
| Male | 315(59·400) | 437(58·400) |  |
| Female | 215(40·600) | 311(41·600) |  |
| Age^※^ | 56·000(46·000–65·000) | 64·500(55·000–72·000) | <0·001 |
| Height^※^ | 168·000(160·000–172·000) | 165·000(160·000–172·000) | 0·028 |
| Weight^※^ | 70·00(61·00–77·00) | 68·00(60·00–75·00) | 0·023 |
| Diabetes duration | 5·000(2·000–11·000) | 10·000(4·000–18·000) | <0·001 |
| **Type of disease** | | | |
| Diabetic nephropathy^※^(%) | | | <0·001 |
| No | 452(92·200) | 473(78·400) |  |
| Yes | 38(7·800) | 130 (21·600) |  |
| Diabetic retinopathy^※^ (%) | | | <0·001 |
| No | 470(95·900) | 516(85·600) |  |
| Yes | 20(4·100) | 87 (14·400) |  |
| Hyperlipidemia (%) | | | 0·994 |
| No | 342(69·800) | 421(69·800) |  |
| Yes | 148(30·200) | 182 (30·200) |  |
| Hypertension^※^(%) | | | 0·010 |
| No | 265(54·100) | 279(46·300) |  |
| Yes | 225(45·900) | 324 (53·700) |  |
| Atherosclerosis^※^(%) | | | <0·001 |
| No | 196(40·000) | 177(29·400) |  |
| Yes | 294 (60·000) | 426 (70·600) |  |
| **Blood routine** | | | |
| White blood cell count^※^ | 6·135(5·050–7·290) | 6·060(5·040–7·340) | 0·483 |
| Hemoglobin | 140·000(131·000–151·000) | 137·000(125·000–148·000) | <0·001 |
| Monocyte absolute value | 0·450(0·380–0·550) | 0·440(0·360–0·560) | 0·711 |
| Eosinophil absolute value | 0·110(0·070–0·190) | 0·110(0·070–0·180) | 0·648 |
| Basophil absolute value^※^ | 0·030(0·020–0·050) | 0·030(0·020–0·040) | 0·015 |
| Mean red blood cell volume^※^ | 10·700(10·100–11·300) | 10·800(10·100–11·500) | 0·026 |
| **Blood Biochemistry** | | | |
| Aspartate aminotransferase | 17·000(14·000–22·000) | 16·000(14·000–21·000) | 0·078 |
| Alanine aminotransferase^※^ | 20·000(14·000–31·000) | 17·000(13·000–24·000) | <0·001 |
| Albumin^※^ | 41·900(39·600–43·800) | 40·800(38·400–43·300) | <0·001 |
| Total bilirubin^※^ | 10·440(7·880–13·790) | 9·850(7·300–12·950) | 0·005 |
| UREA^※^ | 5·345(4·405–6·440) | 5·815(4·705–7·155) | <0·001 |
| Creatinine | 66·600(56·000–77·800) | 66·100(56·000–80·100) | 0·319 |
| Serum bicarbonate | 23·500(22·000–25·200) | 23·600(21·900–25·400) | 0·540 |
| Uric acid | 317·000(269·000–393·000) | 318·000(262·000–377·000) | 0·231 |
| Lipase | 41·000(32·000–55·000) | 39·000(29·500–54·000) | 0·056 |
| Total cholesterol^※^ | 4·585(3·850–5·280) | 4·400(3·555–5·310) | 0·012 |
| Triglycerides^※^ | 1·480(1·020–2·170) | 1·320(0·950–2·020) | 0·008 |
| High–density Lipoprotein cholesterol | 1·210(1·040–1·450) | 1·220(1·020–1·455) | 0·948 |
| Low–density lipoprotein cholesterol^※^ | 2·680(2·150–3·260) | 2·550(1·930–3·255) | 0·025 |
| Glycated hemoglobin^※^ | 8·000(6·800–9·600) | 8·800(7·300–10·500) | <0·001 |
| **Thyroid function** | | | |
| Thyroid stimulating hormones | 1·950(1·300–2·970) | 2·010(1·260–2·970) | 0·735 |
| **coagulation** | | | |
| Prothrombin time | 12·600(12·200–13·000) | 12·700(12·200–13·100) | 0·167 |
| Activated partial thromboplastin time^※^ | 36·000(33·400–38·900) | 35·000(32·800–37·600) | <0·001 |
| **Urine Routine** | | | |
| Hemameba^c^※ (%) | | | <0·001 |
| T1 | 78(14·700) | 149(19·900) |  |
| T2 | 401(75·700) | 444(59·400) |  |
| T3 | 51(9·600) | 155(20·700) |  |
| [Urine](javascript:;) [protein](javascript:;)^d^※(%) | | | <0·001 |
| T1 | 33(6·200) | 117(15·600) |  |
| T2 | 177(33·400) | 287(38·400) |  |
| T3 | 305(57·500) | 332(44·400) |  |
| T4 | 15(2·800) | 12(1·600) |  |
| [Glucose](javascript:;)^e^※ (%) | | | <0·001 |
| T1 | 220(41·500) | 349(46·700) |  |
| T2 | 255(48·100) | 243(48·800) |  |
| T3 | 55(10·400) | 156(20·900) |  |
| **Urine biochemistry** | | | |
| Urine protein quantity^※^ | 39·000(24·000–75·000) | 61·000(32·000–146·000) | <0·001 |
| 24h urine protein quantity^※^ | 80·500(44·500–128·500) | 109·000(60·000–263·000) | <0·001 |
| 24h urine volume^※^ | 2·000(1·400–2·500) | 2·000(1·500–2·450) | <0·001 |
| **Insulin determination** | | | |
| C2/C0^※^ | 3·373(2·426–4·592) | 2·933(1·932–4·125) | <0·001 |
| **Corresponding indicator arithmetic ratio** | | | |
| NLR^※^ | 1·819(1·325–2·567) | 2·127(1·552–2·979) | <0·001 |
| PLR | 112·349(97·892–138·926) | 114·549(89·434–150·217) | 0·145 |
| HOMA–IR^※^ | 2·727(1·626–4·408) | 3·112(1·728–5·436) | 0·011 |

Data are presented as median (interquartile) or number (proportion, %)· ※ is indicated P<0·05·

^a^NDPN: No diabetic peripheral neuropathy; ^b^P value: Mann Whitney test was used for continuous variables and Chi–square test for categorical variables; Hemameba^c^, [Urine](javascript:;) [protein](javascript:;)^d^, [Glucose](javascript:;)^e^: Convert continuous variables to categorical variables, with T representing different categories·
