# Supplementary material for: Study on risk factors of diabetic peripheral neuropathy and establishment of a prediction model by machine learning
Source: BMC Med Inform Decis Mak. 2023 Aug 2;23:146. doi: 10.1186/s12911-023-02232-1 (PMC10394817; doi:10.1186/s12911-023-02232-1)
Supplement: Supplementary file 4 — Additional file 4: Supplementary Table S4. Feature weights in embedded feature selection. [file 12911_2023_2232_MOESM4_ESM.docx]

**Supplementary Table S4.** Feature weights in embedded feature selection.

| **Feature** | **Weight^a^** |
| --- | --- |
| Age | 0.082 |
| alanine aminotransferase | 0.025 |
| albumin | 0.033 |
| total bilirubin | 0.027 |
| urea | 0.031 |
| creatinine | 0.028 |
| Uric Acid | 0.026 |
| total cholesterol | 0.023 |
| glycosylated hemoglobin | 0.039 |
| activated partial thromboplastin time | 0.030 |
| urine protein quantity | 0.045 |
| 24h urine protein quantity | 0.046 |
| Diabetes duration | 0.061 |
| C2/C0 | 0.035 |
| NLR | 0.031 |
| HOMA-IR | 0.024 |

^a^weight: Feature weights for the 16 features selected by the embedded feature selection method.
