# Supplementary material for: Study on risk factors of diabetic peripheral neuropathy and establishment of a prediction model by machine learning
Source: BMC Med Inform Decis Mak. 2023 Aug 2;23:146. doi: 10.1186/s12911-023-02232-1 (PMC10394817; doi:10.1186/s12911-023-02232-1)
Supplement: Supplementary file 5 — Additional file 5: Supplementary Table S5. Hyperparameters combinations in optuna of the 6 models. [file 12911_2023_2232_MOESM5_ESM.docx]

**Supplementary Table S5.** Hyperparameters combinations in optuna of the 6 models

| Model | Parameters | Values |
| --- | --- | --- |
| XGBoost | n_estimators | trial.suggest_int('n_estimators',10,200) |
|  | max_depth | trial.suggest_int("max_depth", 8,20,step=2) |
|  | grow_policy | trial.suggest_categorical("grow_policy", ['depthwise', 'lossguide']) |
|  | learning_rate | trial.suggest_float("learning_rate", 1e-8, 1, log=True) |
|  | gamma | trial.suggest_float("gamma", 1e-8, 1.0, log=True) |
|  | reg_lambda | trial.suggest_float('reg_lambda', 1e-8, 1, log=True) |
|  | reg_alpha | trial.suggest_float('reg_alpha', 1e-8, 1, log=True) |
|  | subsample | trial.suggest_float('subsample', 0.1, 1) |
|  | colsample_bytree | trial.suggest_float('colsample_bytree', 0.1, 1) |
| Logistic Regression | penalty | trial.suggest_categorical("penalty", ['l2','none']) |
|  | C | trial.suggest_float('C',1e-8,1,log=True) |
|  | max_iter | trial.suggest_int('max_iter',100,5000) |
| RandomForest | n_estimators | trial.suggest_int('n_estimators',200,500) |
|  | max_depth | trial.suggest_int("max_depth", 2, 10, step=2) |
|  | min_samples_split | trial.suggest_int("min_samples_split", 2, 10, step=1) |
| Decision Tree | criterion | trial.suggest_categorical("criterion", ['gini','entropy']), |
|  | max_depth | trial.suggest_int('max_depth',8,18) |
| KNN | n_neighbors | 2 |
| Naive Bayes | priors | default |
|  | var_smoothing | default |
